# A Manual of Operative Surgery

**Published:** 1846-10-01

**Authors:** 


I. A Manual of Operative Surgery, based on Normal and Pathological Anatomy. By *J. F. Malgaigne.* Translated from the French by *F. Brittan,* M.D. Small 8vo, pp. 580. London, 1846.

II. Practical Surgery. By *Robert Liston.* Fourth Edition. Octavo, pp. 580. London, 1846.

The publication of the above works furnishes us with an opportunity of drawing our reader's attention to a most important subject, viz. the deficiency of the provisions for teaching Operative Surgery in this country. Not that we feel at all disposed to admit the justice of the qualification, intended by M. Malgaigne as complimentary, that English Surgery, once superior, is still *almost* equal to the French. It is quite so, at least as regards the acquirements and achievements of its chief professors, although we fear we must admit it is not so as regards the diffusion of the improvements brought to pass by these. The progress of operative surgery, as indeed of every other branch of medical science, must be mainly due in this, as in any other, country to the zeal, intelligence, and numbers of a limited class of persons who have sufficient opportunities at command, and sufficient talent and industry to avail themselves of them. To the general mass of practitioners the meeting with operations cannot occur with sufficient frequency to enable them to improve upon the modes of conducting them, or even in regard to those of a capital character, to justify their undertaking them at all. But it is none the less important that they should be fully prepared to meet such as may fall to their lot, some of these indeed occurring under such emergencies as to render other aid unavailable, or being so beset with difficulties and danger as to make a serious call upon the knowledge, manual dexterity, and nerve of the attendant. Mr. Liston justly much insists upon the necessity and importance of young men duly qualifying themselves for this important part of their duties, and strongly animadverts upon its neglect by even those who have had good opportunities of cultivating it.

"The art of operating has, even by many of those in prominent situations, been too lightly considered and too little practised on the dead body. The foundation of the study of the art must be laid in the dissecting-room, and it is only when we have acquired dexterity on the dead subject, that we can be justified in interfering with the living. Many poor creatures have been sacrificed in consequence of the ignorance, carelessness, and self-sufficiency even of scientific professors, who have either despised or neglected the study of surgical anatomy, the consideration of the casualties which may occur during the various operations, and the due education of their fingers. The infliction of unnecessary pain through want of adroitness in the use of instruments, and consequent protraction of the operative procedure—the hazarding in the slightest degree the safety of any one who puts confidence in us, and entrusts to us his life, or of one who, as in public practice, is, by chance, and without the means of appeal, thrown upon our care—cannot by any means be palliated or defended—and is, in point of fact, highly criminal."—*Introd.*

Common humanity will respond to these sentiments, and the truth and judiciousness of the following expressions concerning the operation for Hernia (with which, and the means of arresting hæmorrhage, it is essential that every practitioner should be acquainted), will be generally admitted.

"It is highly desirable that every practitioner of medicine should fully comprehend the nature of abdominal hernia, should be well aware of its diagnostic signs, and be competent to afford relief before the symptoms have become of so urgent a nature as to indicate great and impending danger to life. Cases of hernia are most likely to be treated safely for the patient, and with judgment and skill, by the practitioner who is fully prepared to proceed to the last remedy when circumstances demanding it arise. It has been very truly said, that skill in operating is of the utmost importance in giving the surgeon self-possession: a bad operator will hesitate in the most simple cases, whilst a good and dexterous surgeon, like a man skilful in the use of weapons, will not enter rashly into difficulties, but, being engaged from conviction, will bring himself through with courage. Every young man, then, should endeavour to acquire some degree of dexterity in operating, for that will go far to make him a judicious surgeon." 533.

It must not be supposed that because Mr. Liston is one of the most brilliant operators this or any other country ever produced, he attaches an undue importance to the art in which he has attained so great a celebrity. Throughout his work he seems impressed with the important maxim of dispensing with operations whenever possible, but performing them with dexterity when unavoidable. He even seems to us to go somewhat out of his way in a work intended as a Treatise on Practical Surgery, in detailing the measures that may render them unnecessary; and takes every possible means of simplifying rather than exaggerating their difficulties, or magnifying the amount of skill requisite to surmount these.

But while rendering homage to the spirit in which his work has been executed, and acknowledging the truth of the statements it contains, as to the importance of the due education of the student in the art of operating, we must not forget the position the author occupies in the Profession as one of the Council of the College of Surgeons; and we may fairly inquire whether he and his colleagues have sufficiently interested themselves in this vital and important subject, one too which seems so especially to fall within their province. The Council enjoy a virtual monopoly of the instruction of pupils in surgery in the metropolis, as well as of examination into their acquirements after the completion of their studies; and we wish to ask how those of them who agree with Mr. Liston, as we doubt not all do, in attaching so much importance to operative surgery, can reconcile with their ideas of duty to the public the conferring their diploma upon many men who have received no kind of instruction in this branch of the art, have never practised the operations upon the dead body, and in some instances have never even dissected at all? We acknowledge that proof of proficiency in it could be ill-demanded by the examining Board, who, in their capacity of teachers, are well aware that those who come before them have not had the opportunities for duly qualifying themselves; but we have a right to demand why this body of accomplished surgeons, well acquainted with the wants of the profession, has not bestirred itself for the provision of such opportunity? Of course the main obstacle to the performance of operations upon the dead in London arises from the scarcity of subjects, which, indeed, during the last season, well-nigh amounted to a dearth. But such scarcity is surely by no means of necessary existence in an immense capital like this, when so much smaller places as Paris and Dublin are efficiently supplied; and it can only arise from the faulty provisions of the Act which was passed under the pretence of securing a sufficiency of an essential commodity to the profession, at the same time that it furnished security from outrages heretofore perpetrated. It is not our purpose here to discuss how far and why this Act has proved ineffective in the former of these respects, as the subject, owing to its increasing importance, must, ere long, come more directly under our notice; but we maintain that, when the Council of the College became long since aware that no provision for a thorough surgical education existed, and that numbers (and when we include those who have contented themselves with the Hall Certificate these are great) were entering the profession quite destitute of this, it was incumbent upon them to institute a rigorous enquiry into the cause of, and to apply to the Government for a remedy for, so serious a grievance. And would this body have not been more worthily and more successfully thus employed in labouring to secure to the great mass of their future members (and present ones, for how many would continue their anatomical studies were the means within their reach) the means of effectual education, than in their recent attempt at the unjust elevation of the few at the expence of the many? Who can doubt, had the Council made a strong representation to Government of the imperious necessity of additional facilities for dissection, backed as their course would then have been by the unanimous voice of an approving profession, that long ere this such facilities would have been accorded. At all events they should have made the attempt publicly and boldly, and not through the mere medium of a quiet half-hour's chat in the reception-room of the Home Secretary; and, impressed with the importance of an acquaintance with operative surgery, they should have declared they would pass no candidate who did not manifest it; and thus remove from themselves a serious responsibility, and throw upon others the *onus* of providing additional facilities of study, or submitting to the inconveniences of a paucity of duly-qualified practitioners. For men who have avowed their conviction of the necessity of this acquisition, there can be no excuse whatever for their continuing to admit persons into their body destitute of it.

While the provisions for the pursuit of surgical anatomy continue so defective in this country, we can feel nowise surprised that young men, whose pecuniary means enable them to do so, resort to Paris for the completion of that portion of their education for which their own country disgracefully denies them the facilities. So, too, we have reason to believe the great mass of the French surgeons are far better qualified as regards the operative portion of their art than are the English. Indeed, when we have observed the number of young men who hurry up to our schools for so limited a period—their neglect of the practical portion of their education, either from lack of material or from their being cramped by the dictation of the ill-contrived and impossibility-requiring programmes of authority—and the futile examinations by which their capabilities are tested, but upon the strength of which they are dispersed all over the country as fitted to meet the exigencies of practice—we have arrived at the conclusion, either that the importance of a knowledge of anatomy for the practice of surgery has been much exaggerated by teachers, or that a vast amount of needless suffering is inflicted by incompetent hands upon unfortunate patients, and that knowledge which might have been obtained from the dead is too often acquired at the expense of the living. Those who are aware of the pains-taking with which our best operators have ever continued to keep up and refresh their anatomical knowledge, cannot but wonder that more cases of mal-praxis do not result from the exploits of the ignorant and half-educated.

The vast improvements which have taken place in the science of surgery during the last half-century, whereby so large a number of operations heretofore considered necessary are dispensed with, and others still retained are so much simplified, have doubtlessly disposed many to look upon the acquisition of skill in operative medicine as being of less importance than it was formerly considered to be of, and to bestow proportionally less pains upon it. This is, however, very wrong. Operations even of a dangerous character may yet fall to the lot of every practitioner; and a glance at the table of contents of either of the works we have now under notice forcibly exhibits under what a multitude of circumstances our possession of manual dexterity and anatomical knowledge is of the last consequence to those who consign themselves to our care. It is to be feared that not a few of our students, unable to obtain sufficient opportunities of dissection, or feeling from the nature of the examination they expect to undergo no compulsion to avail themselves of such as may fall to their lot, hope, by the aid of manuals and treatises like the present, to be enabled to meet the exigencies of practice. But we fear such works must be placed in the same category as anatomical plates, rendering like them great assistance to those who have once made themselves practically familiar with the subject, and speaking an unknown language to such as have not done so. There can be no doubt that one of the worst characteristics of this present epoch consists in the endeavour to impart a knowledge of the various branches of medical science and practice by means of manuals and condensations of various kinds, which, whatever may be their effect in enabling ill-educated students to pass imperfect examinations, can never form accomplished practitioners. It is deeply to be regretted that any repulsive encyclopædiacal reading of this kind should have displaced, to the extent it has, the perusal of the instructive and captivating productions of the great writers on medicine and surgery of this and other countries. Well founded as we feel this criticism to be, we are not justified in introducing it in the present article without excepting Mr. Liston's work from its operation. Indeed, its chief fault seems to us, in its possessing scarcely enough of the characters of the manual when we consider it is intended as a guide for young practitioners in surgery. Thus its description both of the precise pathological conditions of parts requiring manual interference and of the modes in which this last is to be rendered, are by no means sufficiently minute and clear to render reference to another work unnecessary; while the book has been expanded and rendered expensive by unnecessarily dwelling upon the general features of the various diseases adverted to, and by the republication of cases which had already appeared long since in the Lancet, and a simple reference to which would have sufficed. Nor do we think the very cursory notices of the various applications of tenotomy, the cure of aneurism by compression, and other interesting topics which have of late excited the attention of the surgical world, by any means sufficient. However, the author of a work which, in so short a space of time has reached its fourth edition, can well afford to disregard more important criticisms than we feel ourselves in a position to offer; and, in fact, we think the popularity which the work has attained is, in the main, fully justified by its great and undoubted merits. As a manual, M. Malgaigne's Operative Surgery has been so long held in esteem on the Continent, that it is very surprising it has not before this been translated. Its descriptions are frequently admirable, and the author's appreciation of the various operative procedures discriminating and sagacious. Treating upon the same department of surgery, it is singular to observe the portions of the subject each has not thought proper to embrace in his plan. Thus, Mr. Liston presents us with no account of the operations upon the teeth or the eye: and M. Malgaigne has no description of the treatment required for fractures and dislocations.

Of works of so comprehensive a character, any detailed notice is out of the question. Moreover, with Mr. Liston's former editions most of our readers are familiar; and we have not been able to discover the "considerable additions" advertized as having been made to the present one. We may, however, advert to one or two topics.

*Bleeding from the Foot.*—We copy M. Malgaigne's account of this simple operation because we think it is far too seldom had recourse to in this country, seeing the great relief it is capable of affording in many affections of the portal system and generative organs, and the ease with which blood may frequently be so obtained, when its abstraction from a vein in the arm is either difficult or impossible.

"The internal saphena vein may be opened in front of the internal malleolus, or the external saphena in front of the external malleolus: but the latter is seldom large enough to be opened when the internal is not.

"The patient, seated in a chair or at the edge of his bed, first places his feet in hot water, until the veins are very apparent: then the surgeon selects the foot, wipes it, rests it on his knee, protected by a napkin, and places the ligature two fingers' breadth above the ankle, moderately tightening it, and securing it with a bow on the opposite side. He then explores the vein, puts the foot again in the hot water, prepares his lancet, retakes the foot, and opens the vessel. Care must be taken not to prick the bone and break off the point of the lancet. If the blood flows in a jet it is caught in a basin, if it only dribbles slowly the foot should again be put in the water. We can then only judge of the quantity by the time or redness of water. When the bleeding is supposed to have been enough, the foot is taken out of the water and wiped, and a compress and figure of eight bandage applied. Care must be taken lest the water be too hot, or the foot plunged in too deeply. It is said that the weight of the column of the water tends to coagulate the blood, which stops the mouth of the opening. On this account the foot should be kept only just covered, and the wound wiped from time to time. It is well also to make the patient move his toes." P. 53.

*Opening Abscesses by Incision.*—M. Malgaigne lays down the following general rules:—

"1. In order to lessen the suffering of the patient, the abscess should, if possible, be incised with one stroke of the knife. 2. It is proper that one extremity of the incision should terminate in the lowest part of the abscess. It has also been recommended to make the incision in the direction of its greatest diameter, and, if possible, parallel to the axis of the body, or well-marked folds of the skin; these two rules are subject to too many exceptions to be strictly preserved. 3. One opening is usually sufficient; it should be large enough to afford a free exit to the pus, if not, it should be enlarged with the bistoury and director. 4. When the abscess is very large, it is better to make several incisions than one of too great extent. 5. When a first incision does not reach the lowest part of the abscess, and when compression will not bring the sides of the sinuses together, recourse must be had to a counter-opening, either by cutting down upon the skin raised upon a director introduced through the first opening, or by retaining the pus at the point where we wish to open it, and incising as for common abscess. 6. When the abscess is prominent and superficial, incision from within outwards is preferred: but when the thickness of the parts prevents this, the incision should be made from without inwards with a straight bistoury. 7. When the abscess is situated under a muscle, it is better to separate its fibres than to cut them; but if this is not compatible with the direction of the incision, they may be cut across." P. 79.

Mr. Liston has some interesting remarks likewise upon this important point in minor surgery. He considers a broad-bladed, sharp-pointed bistoury in a folding handle, and fastened when opened by a spring-catch, as a far more workman-like (a vulgar but favourite mode of expression of his) tool than the abscess-lancet in general use. The knife is to be entered steadily with its blade perpendicular to the surface, and pushed onwards until the lessened degree of resistance allows its point to move freely. The superimposed parts are to be divided by a rapid sawing motion. Sometimes it has to be carried very deeply before the matter can be reached, and which, with due care, may be done safely even in the vicinity of important organs, the vessels and nerves being removed by the morbid accumulations to a much greater distance from the surface than they are wont to be normally. A free and clean opening should at once be made, so as to render squeezing the tender parts, extension of the aperture, or counter-openings unnecessary.

"It ought to be recollected, that an opening of an inch in length, quickly and smoothly made, is attended with no more pain than a coarse and hazardous plunge of an abscess lancet, which, though the inflamed surface may be partially lacerated to some considerable extent, will be found, perhaps, to have barely penetrated the cavity by an aperture of not more than two lines,—the object being, after all, inefficiently fulfilled. The opening must be uniformly made at that part of the abscess which is most likely to be generally dependent; the state of the patient, and his probable position for some time after his little operation, must, with that view, be carefully considered before hand." P. 11.

Occasionally it is better to make more than one opening even at first. Abscesses extending over a large surface can seldom be completely discharged without. This is generally the case with abscess over the ligament of the patella resulting from bruise or inflammation of the bursa. An opening in this case should be at once made upon each side of the limb at the most dependent parts of the abscess. This expedites the cure, and is productive of far less pain than would be produced by subsequent incisions, which the progress of the case might require.

Mr. Abernethy's plan of partially evacuating large chronic abscesses, and temporarily closing the valvular aperture, is now exploded, on account of the violent constitutional irritation it often gave rise to. The opening, on the contrary, should be free and direct, so as to allow of no lodgement of matter, even if a counter-opening be required to prevent this.

"The common and thoughtless practice of squeezing together the sides of suppurating cavities, whether chronic or acute, ought by all means to be discouraged. The patient, it is true, seems to be relieved at the time from a greater load of matter, but in reality much pain and positive injury are thus inflicted. The surfaces so treated are apt, from the mechanical injury, to inflame; the vacuum occasioned is filled by air or by rapid secretion of bloody serous fluid, if not by copious escape of blood from the vessels deprived of their customary support, and torn from their connections. The after discharge is profuse and most offensive, and accompanied by a dangerous excitement of the system, an intense irritative fever, and delirium. Those who have had the opportunity of seeing, under the microscope, the injected lining membrane of the cyst of an abscess, will be little inclined to squeeze together the sides of purulent cavities into which they have made openings. The infinite number of vessels that ramify on the surface, and the layer of lymph by which these are covered, are the means by which the filling up and obliteration of the cavity is effected by nature: by bruising and destroying these, very much pain will be caused to the patient, and the cure will not, in all likelihood, be so rapid."—*Liston*, p. 14.

*Lithotrity and Lithotomy.*—Mr. Liston is one of the few surgeons in this country who have taken much pains to acquaint themselves with the performance and advantages of Lithotrity, and his evidence in its favour is of the more importance, inasmuch as the nearly uniform success which has attended his lithotomy operations might have prejudiced him against other procedures. He says:—

"Until very lately, patients applying to surgeons were constantly recommended to submit to the knife, in order to get rid of stone in the bladder, whatever might be the size of the concretion, or the state of the urinary organs. On the other hand, if he fell into the hands of the professed grinder, no matter what the peculiarities of his case, he was as certain to be subjected to the boring or hammering processes. Now that the merits of both operations are better understood and appreciated—some few surgeons having thought proper to turn their attention to the matter, and study and understand lithotrity as well as lithotomy—patients have a chance of being treated judiciously and conscientiously, and of having that proceeding resorted to which is adapted to their respective cases. I was not slow to adopt the operation of crushing, have always had a favorable impression of it, and have throughout used the same language regarding it, yet I have the credit of being an opponent to lithotrity. I have all along been, and am certainly still, opposed to the abuse of any one operation, by its indiscriminate employment in all cases, and by its being practised by those alone who know no other. It can be employed safely only by those who understand well the healthy anatomy of the urethra and bladder; who are acquainted with their sympathies, vital actions, and pathological changes; and who both understand, and are in the constant habit of treating, derangements of their functions. The operation of lithotrity is applicable to patients above the age of puberty, when the symptoms have not endured very long; when the foreign body is ascertained to be about the size of the one sketched two pages back—measuring six or seven lines, or even more perhaps, say as large as a chesnut;—when the bladder and urethra are in a tolerably healthy and normal condition—as indicated by the power to retain the urine comfortably for several hours, and to pass it in a tolerably free stream; and when the viscus admits of injection and a careful exploration. * * * * * * * *

"The operation of lithotomy must yet continue to be performed on children, and on those of mature age who are so ill-informed or foolish as to permit the stone to attain an inordinate bulk. The concretions in young subjects are generally composed of a very dense substance, the oxalate of lime, in whole or in part; and the urethra is often so narrow as to preclude the application of instruments strong enough to reduce them to fragments. The reasons for giving a preference to incision over crushing, when the stone is large, have been already given.

"This favourable opinion of lithotrity was published in 1840: since when it has been confirmed and much strengthened by ample experience. Of late years, in point of fact, I have scarcely been obliged to have recourse to lithotomy at all in private practice. At the hospital, patients yet present themselves with large stones and bad bladders. Then lithotomy is both a less painful and much more safe operation, as already propounded. During the above period twenty-four patients have been cut, and all have recovered without accident; these patients have been of all ages from two to eighty years of age, and some of them not over favourable subjects. So that, after all, there is not much to find fault with as regards this cruel and bloody 'operation' when carefully set about." P. 500–5.

It is satisfactory to know that Mr. Liston's great success as a lithotomist has been obtained by the use of the simplest apparatus, his incisions being commenced and completed by the same straight knife. He makes his external incision very free, but the internal one is very limited. "It should certainly not extend beyond six or seven lines from the urethra outwards and downwards," which in all ordinary cases will allow the requisite dilatation to be safely made. "The object in following this method, is to avoid all interference with the reflexion of the ileo-vesical fascia from the sides of the pelvic cavity over the base of the prostate gland and side of the bladder. If this natural boundary betwixt the external and internal cellular tissue is broken up, there is scarcely a possibility of preventing infiltration of urine, which must almost certainly prove fatal." An opening of this extent, owing to the elastic and yielding character of the parts, will readily admit the finger, and allow of the extraction of a stone of considerable dimensions. In cases in which, from the corpulence or unmanageableness of the patient, or the size and rigidity of the prostate, the inner opening is executed with difficulty, the blunt gorget may be employed for enlarging the opening into the bladder, but Mr. Liston has never had occasion to use it. The exact position of the stone must be carefully ascertained before using the forceps, and its favorable adjustment within these carefully adjusted by the finger. The use of the forceps is in fact the most difficult part of the operation; "and it is equally the duty of the pupil to practise the seizing and extraction of the stone, as it is for him to study the relative anatomy, so that he may be enabled to cut into the bladder with precision and safety." An important piece of advice this which is much needed, as students invariably occupy themselves with the cutting part of the operation alone. If the surgeon finds upon examination with his finger that the stone is a very large one, he must convert his operation into a bilateral one before attempting to remove it. If his external incision has been sufficiently free and low enough in the perineum it will require no enlargement, and he has only to pass a narrow-bladed, blunt-pointed bistoury along the finger, and make a similar incision on the opposite side of the neck of the bladder, directing the edge of the knife towards the right tuberosity of the ischium. In nineteen cases out of twenty the single lateral incision suffices. When the incisions are placed low, and the deeper ones performed cautiously, there is no danger of hæmorrhage; and of considerably more than 100 operations performed by Mr. Liston, only one old man suffered from hæmorrhage, and that because the diseased condition of the branches of the hæmorrhoidals prevented their contracting when divided. Mr. Liston attributes much of the success of his operations to his practice of introducing through the track of the wound, and securing in the bladder, a gum-elastic tube, which, by facilitating the flow of urine, much lessens the danger of infiltration and its dreadful consequences, and affords an easy means of suppressing any excess of arterial or venous oozing. It need not be retained in children for more than 20 hours, but in old persons of lax fibre it should be kept in for 40 or 50 hours at least.

"The operation of lithotomy, about which so much dread has lately been excited and fomented by interested persons, and which certainly, according to the complicated methods, and more especially with the great apparatus delineated at p. 506, was formidable and not over-successful—if performed in the very simple and easy method recommended, is effected with much less pain than is supposed; it is completed with perfect safety, in a short space of time, and offers very favourable results. It is, however, an operation that never ought to be undertaken without due consideration of all the circumstances that may arise; and the surgeon who undertakes it must have resources within himself sufficient to encounter and overcome difficulties in all the various stages of the proceeding. Were the circumstances of all cases precisely similar as regards the depth and resistance of the parts, the size and consistence of the prostate and of the foreign body, the capacity of the bladder, and the width of the outlet of the pelves, then the operation might always be completed in a given time with certainty, and in the same manner. But it is not so: unforeseen obstacles occur, from first to last, and the operator must make up his mind to proceed in all cases with the greatest caution and deliberation: he must commence with a determination to finish his task safely and well: and he will also accomplish this quickly, when the state of parts is favorable, and nothing unusual intervenes."—*Liston, p.* 518.

The practice of making so small an internal incision is a controverted point with some of the best operators. M. Velpeau, Sir B. Brodie, Mr. Fergusson, and M. Bresciani de Borsa* are advocates for as small incisions as possible into the prostate being made, while the testimony of Cheselden, Martineau, John Bell, Samuel Cooper, and M. Malgaigne is in favour of a free incision. The last-mentioned surgeon thus speaks of the operation, after having described its varieties invented by different operators:—

"After much study and comparison, I have come to the conclusion that but very little influence on the result of the operations, as regards the life or death of the patients, is to be attributed to the proceeding of operation. It is the pain and inflammation that kill the patients after lithotomy; and the most painful causes of these assuredly are the dragging, tearing, and bruising of the tissues; accidents inevitable in all the proceedings of perineal lithotomy, when the calculus surpasses the most moderate proportions, and which, when the patient does not sink under them, cause the most serious infirmities. I have seen several patients, who had been cut by the most skilful operators, the limits of the prostate being respected, who had lost all faculty of having erections and ejaculating. I saw one who, in addition to the absolute loss of his genital functions, could not retain his urine, and was obliged to keep his penis constantly squeezed by a constrictor. Double, triple, and quadruple incisions do not definitely augment the extent or elasticity of the external layers of the prostate (what does this mean?); and, by dividing it still more, they seem to expose it more also to be bruised in the passage of the calculus.

"In my opinion, there is only one way of rendering perineal lithotomy less dangerous, at least as regards the operation itself; it consists in following a precept entirely opposed to that which is generally laid down, viz. in *dividing the prostate freely on one side beyond its limits;* cutting the neck of the bladder and the cellular tissue, if the size of the stone necessitates it—in a word, making so free a passage for the stone that the wound may remain an incision, and not be complicated by contusions and lacerations. As for the external wound, it seems to me that it might be enlarged advantageously if required, by encroaching more or less on the right side of the raphe, to obviate the necessity of approaching too near the sciatic tuberosity. An external incision, bilateral if necessary, and a unilateral incision of the prostate, but with all the necessary extent, is the proceeding to which I give the preference, and which has already been adopted with success by several skilful lithotomists."—*Malgaigne*, p. 529.

*Inguinal Hernia.*—M. Malgaigne, speaking of *Trusses*, says that much consideration has led him to conclude—1. That three-quarters of the hernias are badly supported by any truss applied in the ordinary manner. 2. The English spring is far preferable to the old French one. 3. That moveable pads, in a great number of cases, have some real advantages over the fixed. 4. That in oblique inguinal hernia the pad should press on the course of the canal, and on the internal orifice, without touching the pubis, unless in some exceptional cases. 5. That in direct hernia the pad should be more voluminous, fixed, and resting on the pubis. 6. That hard pads are most suitable for compressing the canal, soft ones for direct hernia. 7. That it is much to be wished that surgeons would pay especial attention to this very important branch of our art, which has been too long

* See his interesting description of his mode of performing lithotomy, by which very great dilatability of the prostate is implied, *Medico-Chir. Review*, April, 1846.

left to bandagists. M. M. believes that, if trusses were better applied in adults, a cure might be effected in them as well as in children. "I have seen an inguinal hernia cured in one month in an old man 68 years of age, by complete obliteration of the canal obtained by means of simple compression." Mr. Liston, in like manner, says, "I have met with repeated instances in which a person, well-fitted with a truss, has been enabled, after a few months, to discontinue its use."

*The Taxis.*—The following rules for employing this, are laid down by the first-named writer.

"1. Evacuate the bladder to augment as much as possible the capacity of the belly. 2. Recommend your patient to breathe freely, without crying out or raising his head—in fact, not to make the least effort. 3. Make at the commencement but slight pressure, so that you may afterwards augment it by degrees, and continue it longer without bruising the hernia. 4. Return first the parts that last protruded. 5. Return them in the same direction they came out in. 6. In certain exceptional cases these general rules fail, and the patients themselves are in the habit of using some special way or means, to which it is better to have recourse. There are three principal proceedings.

"(1). The surgeon seizes the hernia with one hand, in such a way that the palm of the hand presses on the base, and the fingers all around the neck of the tumour. He raises it in this way, and pushes it in the direction of the ring, compressing with his fingers the part next the ring, so as to diminish it to a suitable diameter. (2). He embraces the hernia with one hand or both (according to its volume), exactly applying his fingers on its entire surface, so that if possible no portion may remain uncovered, and presses all parts of the circumference of the tumour towards the centre. The efficacy of this pressure is augmented by, as it were, drawing the tumour out from the belly, and carrying it from one side to the other, compressing it with your fingers, so as to unfold the portion of the intestine contained in the ring, and cause the wind and fæces that engorge the hernia to re-enter the belly. (3). He leaves free the bulk of the tumour, but applies one or two fingers close to the ring on the sides of the hernial sac, and pushes thus across the ring the part immediately next to it. When you have in this way returned a small portion, keep it back with the fingers that pushed it in, and push up another, but with the fingers of the other hand, and so on. It often happens that when the strangulated portion, which formed a sort of plug, is returned, the rest follows easily. * * * * * * * * It remains for us to make known three other modifications which seem to us to deserve serious attention. M. *Ribes* takes a mattress, folded and doubled up, so that the edge of the superior fold passes a little beyond the edge of the inferior one, and the surface of the mattress describes a very oblique plane: one or two bolsters are put under the doubled end of the mattress, to increase this obliquity, and the whole is covered with a sheet. This done, the patient is placed on the bed, with his buttocks on the edge of the mattress, and his thighs stretched out and in a line with his belly, and with his pelvis much elevated, and his diaphragmatic region as low as possible. His head must be supported with a pillow, and he must keep in this position all the time required. Then the taxis is tried; and, between each attempt, a bladder of ice kept on the tumour. *Amussat* gives the patient a similar position, but, in addition, he flexes the thighs to relax the abdominal muscles, and inclines the entire body to the side opposite the hernia; and, lastly, while he is performing the taxis, an assistant keeps up a gentle traction on the belly, trying to draw it to the healthy side, and raises from time to time the parietes of the abdomen, slightly pinching up the skin. But what is more important, M. Amussat continues the taxis during two, three, or four hours, or even more, and has, by these means, reduced hernias that seemed only curable by operation. It is an admirable method, when you are sure the intestine is free from gangrene; but one to which recourse must not be had when the duration of the strangulation, or the rapid progress of unfavourable symptoms, gives a reason to dread this fatal termination. Lastly, M. *Koehler* applies a cupping-glass on the hernia itself, to draw outside a large quantity of the intestine, and especially disengage the strangulated portion. This proceeding has seemed to him to greatly facilitate the reduction."—*Malgaigne*, pp. 421-3.

*The Operation.*—After describing the procedures of other surgeons, M. Malgaigne adverts to his own.

" I make my incision, not on the sac and scrotum, but on the place where the strangulation seems to be situated, prolonging it upwards and downwards to the extent required by the *embonpoint* of the subject, and the size of the hernia. All the tissues are divided in the same way down to the peritoneum; and, as we thus expose and lay bare the vessels, we put them aside, and have nothing to fear from them. If you find the strangulation is caused by a fibrous opening, you need not open the sac, but reduce the hernia. If not, the neck is divided from without inwards, carefully and gently; or, if the stricture appears very strong, you make a small incision in the peritoneum, either above or below the neck, which you then raise upon a director and divide.

" In this proceeding I find the great advantage of being able to see everything as I go on. 2. I reach the strangulation by the shortest way, and with the least possible incision. 3. I leave untouched the scrotum and sac, and consequently am not troubled with the cicatrization and suppuration of a wound, at all events useless. I lately performed it for a large scrotal hernia; the neck of which was situated on a level with the abdominal ring, the sac, which I had respected, though I opened its neck, filled in the first few days with a certain quantity of liquid, which was re-absorbed as the inflammation of the superior wound went down, and the patient was cured without accidents." P. 429.

*Operation without opening the Sac.*—Mr. Liston thus states his opinion upon this important point of practice.

" He will thus cut the resisting tissues to a sufficient extent, in a line parallel to the linea alba : the reduction of the contents of the sac may then be attempted; and, if it prove successful, one great danger of the operation, that arising from exposure of the peritoneal sac, the lowering of its temperature, and the consequent shock upon the system, is avoided. But, even in favourable cases of this variety of hernial protrusion, there are difficulties to be encountered in accomplishing this most desirable object; the constriction is caused by a condensed cellular and fibrous tissue immediately investing, and incorporated with, the serous cyst. The stricture may, in fact, be said to exist very often in the neck of the sac itself, and this must be cut before the contents can possibly be returned. In many cases, the reduction is impeded by adhesions, and by entanglement with omentum : again, when the strangulation has existed any considerable time, it is desirable to ascertain the precise condition of the protruded parts, and to consider, after careful and actual inspection, whether they should be returned or not." P. 551.

When treating of *Femoral Hernia,* he thus expresses himself :—

" He may now try to reduce the contents of the sac by pressure: taking especial care not to push back sac and all—rather a serious accident. This plan was advocated strongly, long ago, by Petit and Monro secundus, and has lately been revived and practised by Mr. Key, Mr. B. Cooper, Mr. Luke, and others. I have fortunately succeeded in effecting this object in a considerable number of instances, within these few years: and it is a proceeding which I should most strenuously advise the adoption of, when nothing contra-indicates it. The attempt can do no harm: it causes little or no delay, and, if it is not successful, the sac, after all, is opened, and the operation completed. If it does prove successful, the surgeon's mind is relieved of an uncommon load of anxiety." P. 558.

*Dilatability of the Vagina.*—M. Malgaigne states, in the following passage, the varying degree of this according to age:—

"The dimensions of the vaginal orifice are very variable. It is important to know that in young women it is exceedingly dilatable; it is less yielding in adults; and after the cessation of the menses its rigidity goes on increasing, so that, at a very advanced age, instead of feeling at this orifice a supple ring, yielding under the fingers, we find it hard and splitting at the slighest effort made at overcoming its resistance. It then sometimes scarcely admits the little finger, and the vagina itself, instead of offering its usual rugæ, presents polished walls and a very constricted capacity. The result is that, in young women, however narrow the orifice may appear, we may confidently rely on its dilatability; that, in adults, we must not place so much reliance on it, and must use a speculum not much larger than the apparent capacity of the orifice; and, lastly, that at a later period, we should be more reserved in employing the speculum at all; proceed with gentleness and caution, in order to avoid lacerations, which cicatrise with difficulty; and use only a very small speculum. Lisfranc has found it necessary to prepare the parts, during eight or ten days, by dilating them with prepared sponge." P. 543.

We must here conclude our extracts, and can only wish a continuance of success to two such excellent works.

